# Rationale for Determining the Functional Potency of Mesenchymal Stem Cells in Preventing Regulated Cell Death for Therapeutic Use

**DOI:** 10.5966/sctm.2016-0289

**Published:** 2016-10-11

**Authors:** Abderrahim Naji, Narufumi Suganuma, Nicolas Espagnolle, Ken‐ichi Yagyu, Nobuyasu Baba, Luc Sensebé, Frédéric Deschaseaux

**Affiliations:** ^1^ Center for Innovative and Translational Medicine, Kochi Medical School, Kochi University, Kochi, Japan; ^2^ Department of Environmental Medicine, Kochi Medical School, Kochi University, Kochi, Japan; ^3^ STROMALab, INSERM U1031, EFS Pyrénées‐Méditerranée, Université de Toulouse, Toulouse, France; ^4^ Science Research Center, Division of Biological Research, Life Sciences and Functional Materials, Kochi Medical School, Kochi University, Kochi, Japan

**Keywords:** Mesenchymal stem cells, Cell death, Functional potency, Cellular therapy, Degenerative disorder, Inflammatory disorder, Clinical translation, Selection technologies

## Abstract

Mesenchymal stem (stromal) cells (MSCs) are being investigated for treating degenerative and inflammatory disorders because of their reparative and immunomodulatory properties. Intricate mechanisms relate cell death processes with immune responses, which have implications for degenerative and inflammatory conditions. We review the therapeutic value of MSCs in terms of preventing regulated cell death (RCD). When cells identify an insult, specific intracellular pathways are elicited for execution of RCD processes, such as apoptosis, necroptosis, and pyroptosis. To some extent, exacerbated RCD can provoke an intense inflammatory response and vice versa. Emerging studies are focusing on the molecular mechanisms deployed by MSCs to ameliorate the survival, bioenergetics, and functions of unfit immune or nonimmune cells. Given these aspects, and in light of MSC actions in modulating cell death processes, we suggest the use of novel functional in vitro assays to ensure the potency of MSCs for preventing RCD. Such analyses should be associated with existing functional assays measuring the anti‐inflammatory capabilities of MSCs in vitro. MSCs selected on the basis of two in vitro functional criteria (i.e., prevention of inflammation and RCD) could possess optimal therapeutic efficacy in vivo. In addition, we underline the implications of these perspectives in clinical studies of MSC therapy, with particular focus on acute respiratory distress syndrome. Stem Cells Translational Medicine *2017;6:713–719*

**Significance Statement**

Most studies of mesenchymal stem (stromal) cells (MSCs) focus on their anti‐inflammatory, trophic and differentiation abilities, but their ability to prevent regulated cell death (RCD) remains undefined. However, this last function could explain both the regenerative and anti‐inflammatory therapeutic effect of MSCs observed in preclinical and clinical studies. The present report reviews the role of MSCs in preventing RCD, with implications for enhancing their therapeutic efficacy in the clinic. Development of in vitro assays to assess MSC functional potency in preventing RCD is suggested and criteria for selecting MSCs for therapeutic use are proposed. Furthermore, in vivo biomarkers of RCD that can be used for prompt evaluation of the therapeutic effects of MSCs are suggested.

## Introduction

**Figure 1:**
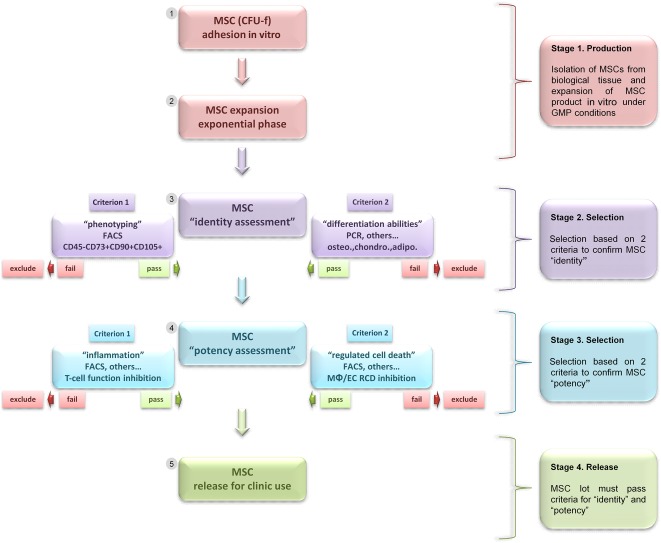
Schematic diagram summarizing the concept of MSC selection based on identity and double functional potency for preventing inflammation and RCD before use as therapy. This schematic shows four essential stages, from isolation to release of MSC product for use in clinic. Stage 1: optimal methods for MSC isolation, expansion, and production by GMP with severe control in cell sterility and genetic stability. Stage 2: selection of MSCs based on two criteria, phenotype and potential for differentiation, for assessing MSC “identity” in vitro. Stage 3: selection of MSCs based on two criteria, inhibition of inflammation and inhibition of RCD, for assessing MSC “potency” in vitro. Stage 4: for approval of MSCs for therapy and monitoring of in vivo actions of MSCs. Abbreviations: adipo., adipocytes; CFU‐f, colony‐forming unit fibroblast; chondro., chondroblasts; EC, epithelial cell; FACS, fluorescence‐activated cell sorting; GMP, Good Manufacturing Practices; MΦ, macrophage; MSC, mesenchymal stem (stromal) cell; osteo., osteoblasts; PCR, polymerase chain reaction; RCD, regulated cell death.

Mesenchymal stem (stromal) cells (MSCs), in humans, are principally derived from bone marrow and adipose tissues in adults and in neonatal tissues from umbilical cord blood and placenta [1, 2, 3]. Regardless of their origin, in vitro‐expanded MSCs possess a common phenotype and share mutual biological properties [4, 5, 6, 7, 8]. However, we lack specific biomarkers to distinguish MSCs phenotypically and exclusively in vivo or in MSCs expanded in vitro. This situation is further complicated by the fact that in vitro‐expanded MSC cultures are not derived from a single clone but rather several fibroblastic colony forming units [9, 10] with probable functional heterogeneities [8, 11]. To address this complexity, researchers use a combination of cell surface markers [7, 8] that are often associated with functional assessment of MSCs in differentiating into osteoblasts, chondroblasts, and adipocytes to confirm the MSC identity [8] (Fig. 1).

Today, MSCs are under intense clinical investigation for regenerative medicine because of their differentiation and trophic abilities [12, 13, 14] and for treatment of inflammatory diseases because of their immunosuppressive properties [15, 16]. MSCs delivered in vivo can home to inflammatory sites [17, 18] and produce anti‐inflammatory and growth factors; therapeutic effects have been demonstrated in preclinical and clinical studies of various disorders [19, 20]. Hence, the clinical use of MSCs for treating severe degenerative and inflammatory diseases lacking appropriate treatments is expected to increase exponentially [8].

Substantial efforts have been undertaken by the translational community to standardize methods for producing, selecting, and using MSCs in the clinic [5, 6]. Notably, general guidance has been proposed for developing in vitro assays for selecting MSCs with potent therapeutic ability based on functional criteria [20, 21]. These assays require identifying MSC functions to predict clinical efficacy [6]. Some clinical observations have confirmed the relevance of in vitro assays to measure anti‐inflammatory MSC potency, which was found consistent with in vivo effects [21]. Challenges remain in improving and using pertinent functional in vitro assays to identify MSCs with bona fide optimal efficiency in vivo [5, 6]. Thus, the ability of MSCs to prevent cell death processes could be tested in vitro to identify functional MSCs for clinical use.

## Regulated Cell Death as a Therapeutic Target

Emerging evidences indicate a critical role for regulated cell death (RCD) in the pathogenesis of various diseases [22]. By definition, RCD is opposite to accidental cell death (ACD), whose effects are often identified as necrosis [23] (Tables 1, 2). ACD results from sudden trauma and occurs in an uncontrolled manner [23]. Nonetheless, ACD occurring in cells and through the release of intracellular content might trigger RCD in bystander cells [23]. RCD includes several processes [24, 25], among which the most distinct are apoptosis, necroptosis, and pyroptosis [23] (Tables 1, 2). Thus, RCD is caused after cells sense danger or inflammatory mediators, in sterile or nonsterile conditions, which has implications for the pathogenesis of degenerative and inflammatory disorders [22, 24, 25, 26, 27, 28].

**Table 1:** Features of RCD and ACD with the role of MSCs in preventing RCD in terminally differentiated third‐party cells

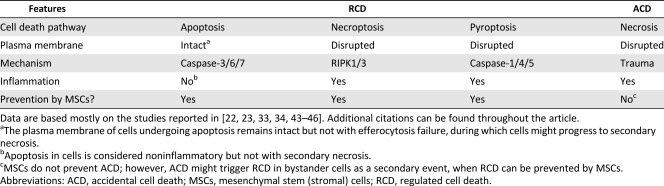

**Table 2:** MSC prevention of RCD processes occurring in terminally differentiated parenchymal, stromal, and immune cells

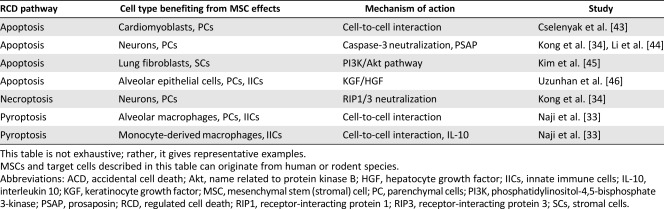

The RCD processes differ by their molecular triggers, molecular pathways engaged and mode of execution [23]. Apoptosis has been considered a programmed cell death (PCD) both during physiological and pathological processes. The term “PCD” is now preferred to indicate cell death from physiological processes, such as during development and maintenance of tissue homeostasis [29]. However, when cell death occurs during pathological conditions, RCD rather than PCD appears more appropriate [23, 25, 29]. Apoptosis is executed via a mechanism involving caspase‐3/6/7 and results in cell death without plasma membrane rupture [22]. With disrupted plasma membrane, apoptosis might culminate in secondary necrosis [24, 30, 31]. Thus, apoptosis can be considered nonimmunogenic but not occurring in particular pathological conditions [26, 30, 31], whereas RCD such as necroptosis and pyroptosis are intrinsically immunogenic [24].

Necroptosis is mediated by a mechanism that depends on receptor‐interacting protein kinase 1/3 and mixed lineage kinase‐like protein, whereas pyroptosis is executed in cells by a mechanism involving caspase‐1/4/5 and gasdermin D [24, 26, 27]. Both necroptosis and pyroptosis conclude with a rapid rupture of the plasma membrane, release of intracellular content and often with harmful consequences [24, 27]. Hence, RCD can be detrimental because it can sustain inflammation, tissue damage, and loss of function of the affected organ [22, 28]. Furthermore, exacerbated RCD can cause inflammation, and intense inflammation can elicit RCD, with, in all cases, pathological consequences [22]. Therefore, targeting RCD in addition to inflammation is needed to improve the efficacy of existing anti‐inflammatory therapeutics [22, 28, 32].

## Brief Insights Into the Prospective Mode of Action of MSCs in Preventing RCD

MSCs are known to improve cell survival and prevent apoptosis, necroptosis and pyroptosis (Tables 1, 2) occurring in various parenchymal or nonparenchymal cells and immune cells under unfavorable conditions [19, 33, 34, 35]. Mechanistically, MSCs are thought to promote cell survival via the secretion and paracrine actions of various cytokines and growth factors [20, 36]. They may also promote survival, bioenergetics, and functions of distressed cells, by mitochondria transfer through tunneling nanotubes (TNT), or microRNA/protein transfer through extracellular vesicles [37, 38, 39, 40]. The mechanism may involve gap‐junction communication via connexin 43 between MSCs and unfit cells [38, 41]. Consistently, mitochondrial transfer from MSCs to immune cells occurs in vivo and results in enhanced cell survival, phagocytic activity, and antimicrobial effects in preclinical models of acute lung injury and acute respiratory distress syndrome (ARDS) [38, 39]. The mechanisms MSCs use to achieve improved survival, bioenergetics, and functions of unfit cells are diverse and sophisticated and may reflect their vital importance, such as preventing RCD. Of note, TNT‐mediated transfer of mitochondria from healthy to apoptotic neuroblastic PC12 cells can reverse apoptosis, with implications for the survival mechanisms of damaged cells [42]. By comparison, this proposes that transfer of mitochondria from MSCs to distressed cells through a TNT‐dependent mechanism might prevent the execution of RCD.

Therefore, innovative therapeutic interventions should simultaneously target RCD and inflammation to optimize cure [22]. The abundant success of MSC therapy in certain degenerative and inflammatory disorders, observed in preclinical and clinical studies, might be because of the intrinsic properties of MSCs to simultaneously modulate RCD and inflammation. Further dissecting the mechanisms MSCs use to prevent RCD is fundamental, but the use of such functional attributes as selection criteria for MSCs intended for therapy is of immediate practical importance for the clinic.

## MSC Function to Modulate RCD as Criteria for Therapeutic Use

The antiapoptotic properties of MSCs toward immune and nonimmune cells have been demonstrated in some contexts [35, 36, 43, 44, 45, 46]. Emerging studies suggest that MSCs can inhibit RCD such as necroptosis [34], and we recently showed that MSCs could prevent pyroptosis in macrophages [33]. We focused on the pathogenesis of severe occupational lung diseases such as interstitial lung disease and pulmonary alveolar proteinosis, which could involve pyroptosis of lung macrophages caused by inhalation of inorganic particles [33]. This pyroptosis is characterized by the production of inflammatory cytokines and cell death by cytolysis, events depending on the inflammasome NACHT, LRR, and PYD domain‐containing protein 3–apoptosis‐associated speck‐like protein containing a CARD–Caspase‐1 (NLRP3‐ASC‐Caspase‐1) [33]. Blockade of inflammatory pathways with pharmacological inhibitors such as dexamethasone and genetic knockdown of essential inflammasome protein components (i.e., NLRP3 or ASC) reduced the production of inflammatory cytokines but were ineffective in preventing cell death. However, coculture of MSCs with macrophages undergoing pyroptosis resulted in both inflammation and cell death inhibition [33].

Therefore, we suggest that to optimize the efficiency of MSC therapy, the ability of MSCs to prevent RCD should be evaluated by in vitro functional assays before the cells are used in clinical interventions (Fig. 1). The assays can be established rapidly and suitably in conventional biology laboratories (Tables 3–5). These functional assays should be implemented by coculturing MSCs with cells of innate immunity, including macrophages and epithelial cells, because RCD in innate immune cells are likely responsible for triggering an exacerbated inflammatory response, such as in sepsis [22]. Thus, macrophages and epithelial cells, challenged with specific cell death inducers, can be cocultured with MSCs at varying cell ratios to estimate the ability of MSCs to modulate RCD. These in vitro functional assays can be used to measure markers of cell death in cells or supernatant (Table 4) within hours [33]. As well, they can allow for quantifying pro‐ and anti‐inflammatory cytokines (i.e., tumor necrosis factor α and interleukin 10) released in the supernatant in assessing MSC function to modulate RCD and inflammation [33]. To further compare the MSC potency of various products to modulate RCD, MSCs should be tested in dilution series with limiting dilution analysis (LDA) [47] to measure the amplitude of potency of a given MSC culture in preventing RCD. Hence, LDA established for each MSC product might help estimate the MSC frequency with actual function to prevent RCD to predict the MSC therapeutic benefit in vivo. However, these functional assays must be accompanied by in vitro evaluation of the MSC anti‐inflammatory potency for cells of adaptive immunity, such as T cells [48]. The selection of MSCs based on in vitro functional criteria for modulating both RCD and inflammation of innate and adaptive immune cells might lead to an optimal therapeutic effect in vivo (Tables 3–5).

**Table 3:** Evaluation of MSC potency based on two functional criteria: inflammation and RCD

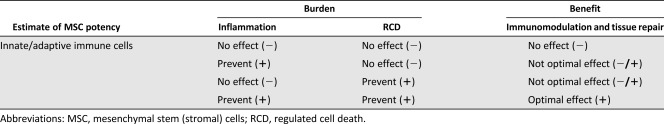

**Table 4:** Evaluating RCD and ACD in vitro with specific RCD biomarkers

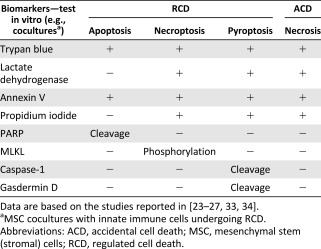

**Table 5:** Evaluating RCD and ACD in vivo with specific biomarkers

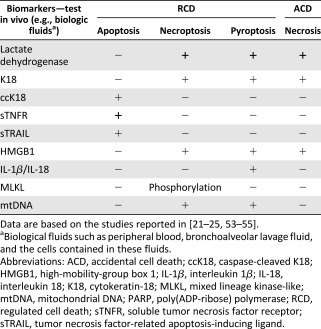

Because the therapeutic effects of MSCs often result from multiple pathways, with or without redundant actions [8], the in vitro potency of MSCs must be assessed in terms of two functional criteria to ensure the optimal in vivo effect. Assessing the MSC potency to prevent both RCD and inflammation, with the assumption that both functions can be determined by independent mechanisms, is critical to ensure the optimal therapeutic effect of MSCs, and particularly for diseases in which cell death is closely related to inflammatory processes, such as ARDS or other devastating disorders [22, 32]. Studies have suggested that the systemic administration of MSCs in preclinical ARDS models improves respiratory conditions [39, 49]. Recently, a phase 1 clinical trial demonstrated the safety and tolerability of intravascular infusion of allogeneic MSCs in nine patients with ARDS [16, 37]. A phase 2 clinical trial in progress [20, 50] is assessing the clinical efficacy of MSC infusion in patients with moderate to severe ARDS [50]. Therefore, selecting MSCs intended for use in treatment of ARDS has clinical relevance in terms of the in vitro potency to modulate both RCD and inflammation.

## Clinical Relevance for Identifying MSCs With Optimal Therapeutic Actions

Indeed, RCD represents a therapeutic target for attenuating both tissue damage and inflammation in various disorders [22] such as ARDS [49]. ARDS represents severe lung injury, a serious and life‐threatening condition that often results from intense trauma, pneumonia infection or sepsis [49]. The pathogenesis of ARDS is characterized by diffuse alveolar damage complicated by intense inflammation [51]. Diffuse alveolar damage is associated with rapid and massive myeloid and epithelial cell death, which is detected by molecular markers such as activated caspases and cleavage of cytokeratin 18 (K18) [21, 49]. Hence, in advanced‐phase clinical trials, the MSC potency in preventing RCD in myeloid and epithelial cells could be evaluated as supplementary selection criteria for MSCs intended for patients with ARDS. This suggestion is motivated by patients with ARDS being particularly affected by intense cell death and inflammation within the lung parenchyma [51]. Furthermore, molecular markers of RCD should be tested in vivo (Table 5) to measure the beneficial effects of MSC adoptive transfer, as an integral part of monitoring MSC therapy, especially for patients with ARDS.

A study by Leblanc and colleagues [21] showed improvement with MSC infusion in severe cases of ARDS, with resolution of respiratory, hemodynamic, and organ failure [21]. These improvements were associated with decreased levels of markers of inflammation. Moreover, the authors evaluated in vitro the immunomodulation potency of the MSCs used. The in vitro potency assays included functional assays for determining the anti‐inflammatory properties of MSCs and proteomic analysis of both MSCs and extracellular vesicles released by MSCs. Encouraging results were observed in two patients with ARDS who received an intravascular infusion of MSCs on a compassionate basis [21]. In these two cases, adoptive transfer of MSCs demonstrated that the in vivo actions of MSCs agreed with most of the MSC actions measured in vitro [21].

Improvements in patients with ARDS who received adoptive transfer of MSCs were associated with a rapid decrease in levels of markers of cell death [21]. Significantly, Leblanc and colleagues analyzed bronchoalveolar lavage fluid (BALF) for monitoring molecular makers of apoptosis and necrosis of alveolar epithelial cells. The analysis of cell death in BALF was based on detection of epithelial apoptosis by measuring caspase‐cleaved K18 and other forms of cell death with features of necrosis, detected by measuring uncleaved K18 [21]. The results revealed a rapid decrease in both apoptosis and necrosis of lung epithelial cells, assessed within only few hours after the adoptive transfer of MSCs in patients [21]. This finding might indicate a sequential mechanism of the MSC action, the first effect being to home to the site of tissue damage, to prevent RCD, before or concomitant with the assessable action of MSCs in modulating inflammation.

Thus, RCD biomarkers could be measured to monitor and rapidly predict the outcomes of a given MSC treatment in patients with ARDS. This analysis is crucial to readily evaluate the response of the intervention in patients and could be used to adapt and appropriately improve the treatment. Leblanc and colleagues suggested that MSCs have therapeutic efficacy for ARDS [21]. Furthermore, the authors demonstrated the advantage of in vitro assessment of the MSC anti‐inflammatory potency while providing critical molecular insights into the processes of cell death as pertinent in vivo biomarkers [21]. Thenceforth, such assessments appear critical in order to rapidly monitor and evaluate the therapeutic effects of MSCs.

## Conclusion

MSCs are remarkable from therapeutic perspectives, given the ease with which we can obtain a significant number of genetically stable MSCs and the number of diseases that can be treated because of the intrinsic properties of MSCs [36]. Today, MSCs are used in advanced‐phase clinical trials of therapy to inhibit the degenerative and inflammatory processes in various disorders [6, 14, 36]. Thus, we increasingly need to standardize, optimize, and ensure the success of MSC therapy in such advanced‐phase clinical trials [5, 6, 9, 13, 14, 20, 21, 48, 50, 52]. The challenges and perspectives lie in implementing appropriate functional assays in vitro that could assess the therapeutic potential of MSCs intended for clinical use. To this end, the efforts of the translational community have focused on providing release criteria for MSCs based on their anti‐inflammatory function, usually toward T‐cell activation and proliferation, in vitro [5, 6, 48]. In this review, we suggest that in addition to developing easy‐to‐use and rapid functional assays for MSCs, we should develop assays to evaluate their ability to modulate RCD and in particular innate immune cells such as macrophages and epithelial cells. However, functional assays for MSCs in modulating RCD of other cell types, such as parenchymal cells or organ‐specific cell subtypes, could be applied; pertinent target cells should be identified according to a known pathogenesis implying RCD for a given disease. In addition, we suggest monitoring RCD biomarkers in patients, including specific markers for apoptosis, necroptosis, and pyroptosis, because these RCD have a direct effect on the pathogenesis of a number of diseases [22, 23, 28]. Of note, RCD may not be relevant in the pathogenesis of all diseases treated with MSCs, in which case other pertinent markers should be evaluated. Nonetheless, targeting both inflammation pathways and RCD pathways as therapeutic objectives might help improve MSC treatments intended for degenerative and inflammatory diseases. The assessment of the potency of MSCs in modulating both inflammation and RCD in vitro and the monitoring of both inflammation and RCD biomarkers in vivo [23, 25, 53, 54, 55] would certainly benefit patients receiving MSC therapy, particularly those with ARDS currently in advanced‐phase clinical trials [20, 21, 39, 50, 51].

## Author Contributions

A.N., conception and design, figure/table design, manuscript writing, final approval of manuscript; N.S., N.E., K‐i.Y., and N.B.: manuscript writing, final approval of manuscript; L.S. and F.D.: conception and design, manuscript writing, final approval of manuscript.

## Disclosure of Potential Conflicts of Interest

The authors indicated no potential conflicts of interest.
